# Impact of Longitudinal Social Support and Loneliness Trajectories on Mental Health during the COVID-19 Pandemic in France

**DOI:** 10.3390/ijerph182312677

**Published:** 2021-12-01

**Authors:** Sandy Laham, Leticia Bertuzzi, Séverine Deguen, Irwin Hecker, Maria Melchior, Martina Patanè, Irene Pinucci, Marit Sijbrandij, Judith van der Waerden

**Affiliations:** 1 Social Epidemiology Research Team, Institut Pierre Louis d’Épidémiologie et de Santé Publique, Sorbonne Université, INSERM U1136, 75012 Paris, France; sandylaham@gmail.com (S.L.); leticia.bertuzzi@iplesp.upmc.fr (L.B.); severine.deguen@ehesp.fr (S.D.); irwin.hecker@iplesp.upmc.fr (I.H.); maria.melchior@inserm.fr (M.M.); 2 Department of Environmental and Occupational Health, EHESP School of Public Health, 35043 Rennes, France; 3 World Health Organization Collaborating Center for Research and Dissemination of Psychological Interventions, Department of Clinical, Neuro- and Developmental Psychology, Amsterdam Public Health Institute, Vrije Universiteit, 1081 HV Amsterdam, The Netherlands; m.patane@vu.nl (M.P.); irene.pinucci@uniroma1.it (I.P.); e.m.sijbrandij@vu.nl (M.S.)

**Keywords:** COVID-19, social support, loneliness, longitudinal, mental health

## Abstract

(1) Background: Little is known about how the COVID-19 pandemic has impacted social support and loneliness over time and how this may predict subsequent mental health problems. This study aims to determine longitudinal trajectories of social support and loneliness in the French general population during the first year of the COVID-19 pandemic and study whether variations in these trajectories are associated with symptoms of depression and anxiety; (2) Methods: Analyses were based on data from 681 French participants in the international COVID-19 Mental Health Study (COMET) study, collected at four periods of time between May 2020 and April 2021. Group-based trajectory modelling (GBTM) was used to determine social support and loneliness trajectories. Associations between the identified trajectories and symptoms of depression and anxiety, measured with the Patient Health Questionnaire (PHQ-9) and Generalized Anxiety Disorder scale (GAD-7), were tested through multivariate linear regression models; (3) Results: Social support trajectories revealed four stable groups: ‘poor’ (17.0%), ‘moderate’ (42.4%), ‘strong’ (35.4%) and ‘very strong’ (5.1%). Loneliness trajectories also identified four groups: ‘low stable’ (17.8%), ‘low rising’ (40.2%), ‘moderate stable’ (37.6%) and ‘high rising’ (5.0%). Elevated symptoms of depression were associated with poor social support as well as all identified loneliness trajectories, while high levels of anxiety were associated with moderate stable and high rising loneliness trajectories; (4) Conclusions: High and increasing levels of loneliness are associated with increased symptoms of depression and anxiety during the pandemic. Interventions to address loneliness are essential to prevent common mental health problems during the pandemic and afterwards.

## 1. Introduction

In Europe, France has been one of the countries that was particularly affected by the spread of COVID-19. Up until July 2021, there were more than 6.3 million COVID-19 cases and more than 110,000 deaths in France [1]. To counter the spread of the virus, the French government declared three national lockdowns: 17 March–11 May 2020, 28 October–15 December 2020, and 3 April–3 May 2021. The majority of these lockdowns mandated the closing of schools, universities, public spaces and favored stay-at-home measures except for vital needs [2,3,4]. In between lockdowns, the French population had to comply with strict sanitary measures including wearing masks, social distancing, remote working and various curfews [5].

While significant public health efforts have focused on protective and treatment measures aiming to battle the spread of the disease, the potential impact of the pandemic on mental health within the population also requires attention. Previous pandemics have been shown to negatively impact the population’s mental health, whether from fear of the disease itself, the impact of the imposed sanitary measures (like quarantine and social distancing) or the impact on the economy [6,7]. Likewise, evidence is emerging for the current COVID-19 pandemic. Globally, increased prevalence rates have been reported for mental health problems [8,9,10,11,12,13]. In France, ongoing cross-sectional data collected by the French national public health agency indicate that the prevalence of both anxiety and depressive symptoms reached a peak of 22.7% in February 2021, compared to 13.5% and 9.8%, respectively, in 2017 [14].

In general, resilience factors are protective characteristics that may shield a person against unfavorable outcomes during adversities [15]. Social support is a resilience factor which by definition refers to accessible support provided by an individual’s social network and plays a major role in protecting from mental illnesses [15,16,17]. It is a multi-faceted concept and while there is no consensus yet regarding its components, it can generally be divided into two dimensions: Structural and functional [18]. While not much research has been conducted exploring the role of social support on mental health problems in the general population during the COVID-19 pandemic, a systematic review has shown that it was protective against traumatic stress, burnout and anxiety in healthcare workers [19]. Furthermore, a five-month longitudinal study in the UK, where online data collection started in parallel with the first lockdown in March 2020, self-reported that sufficient social support was associated with a faster decrease in symptoms of depression and anxiety over time [20]. Another longitudinal study conducted in the US reported increased depression in individuals with low perceived social support [21].

While social support might contribute to a person’s resilience to mental health problems, loneliness could increase this risk. Loneliness is defined as a distressing emotion that comes with the belief that one is socially isolated [22]. Importantly, loneliness is not the equivalent of solitude or being alone, people can live relatively solitary lives and not feel lonely, or they can live abundant social lives yet still feel lonely [22]. Loneliness is a serious public health threat associated with negative physical and mental health outcomes, with several studies reporting an association between loneliness and depression [23,24,25] and anxiety [12]. During the COVID-19 pandemic, an increase in loneliness and inverse association with mental health outcomes has been reported as well. A longitudinal study in four European countries (including France) revealed increased loneliness during the pandemic specifically among young adults and those with a history of mental illness [26]. A study during the third week of lockdown in the United States has shown that loneliness was linked to higher levels of depression and suicide [27]. Another cross-sectional study in the early phases of the pandemic in Spain found that, among other risk factors, loneliness was the strongest predictor of depression and anxiety [28].

The COVID-19 pandemic and the accompanying sanitary measures provide a specific context in which to study the association between social support, loneliness and subsequent mental health outcomes. The current literature on the effect of the pandemic on mental health is mostly based on cross-sectional designs or on specific groups (e.g., healthcare workers), which does not allow to observe changes in social support and loneliness for the general population as the pandemic persists. Few studies have examined how social support and loneliness evolved during the first year of the pandemic as well as their associations with mental health problems, allowing for the development and implementation of mental health policies and interventions in the context of the crisis. Thus, the overall aim of this study is to determine longitudinal trajectories of social support and loneliness in France during the COVID-19 pandemic and to examine whether variations in these trajectories are predictive of adverse mental health outcomes, particularly, symptoms of depression and anxiety.

## 2. Material and Methods

### 2.1. Study Design

The COVID-19 Mental Health Survey (COMET) study is an international, online longitudinal survey aimed at evaluating the course of mental health symptoms during the COVID-19 pandemic and the identification of the individuals who are at risk or resilient to these symptoms. The COMET consortium includes participants from 14 countries (The Netherlands, Italy, Switzerland, Turkey, Spain, Germany, France, United Kingdom, Sweden, South Africa, Indonesia, China, Australia and the United States). Participants were recruited in May 2020 through a snowball sampling strategy using university mailing lists and different social media platforms. Inclusion criteria for participation in the study were: (a) Being 18 years of age or older; (b) having an adequate command of one of the study languages (Dutch, English, German, Italian, French, Swedish, Turkish, Mandarin or Bahasa Indonesian); (c) provide the online informed consent. Before engaging in the study, participants were given information about the study and its objectives and an informed consent from their part was provided through a secure web link before starting the survey. Participation was voluntary and participants were free to withdraw from the survey at any time. Additionally, participants were compensated with an entry into a draw for one of ten “50 euros” vouchers. In total, 8084 participants were recruited for participation in the first data wave.

Included participants were invited to complete a Computer Aided Web Interviewing (CAWI) survey containing validated questionnaires on, among others, depression, anxiety, PTSD, substance use, loneliness, coping, social support, contamination fear, social value orientations as well as questions on socio-demographic factors during the COVID-19 pandemic. Questionnaires were available in the languages spoken in the participating countries. After the first data wave (4 May–7 July 2020), participants were invited to contribute to three additional data collection waves, that took place in 4 September–5 October 2020, 7 December 2020–10 January 2021, and 19 March–23 April 2021. For the specific purpose of this study, we will only use the data from participants who indicated during the first data collection wave to be residing in France. Initially, 681 French participants were recruited, with n = 442, n = 441 and n = 424 participating in the follow-up waves.

The COMET study was approved by the ethical review board of the Faculty of Behavioral and Movement Sciences of the Vrije Universiteit Amsterdam (VCWE-2020-077), and the French contribution to the COMET consortium is in accordance with the Règlement Général sur la Protection des Données (RGPD) and the Informatique et Libertés law. Personal data are protected according to EU and national laws.

### 2.2. Measures

#### 2.2.1. Social Support

At each data wave, social support was measured using the Oslo Social Support Scale (OSSS-3) [18]. This scale determines the level of social support based on three questions scored on a four to five-point scale. The overall OSSS-3 score ranges from 3–14, with higher scores being indicative of higher levels of social support. Categories usually applied are 3–8 = poor support; 9–11 = moderate support; and 12–14 = strong support [18].

#### 2.2.2. Loneliness

At each data wave, feelings of loneliness were measured with a single item question (“Do you feel lonely?”) thus providing insight into participants’ subjective feeling of loneliness. Scores range from 1–5 with a score of 1 = never, 2 = rarely, 3 = sometimes, 4 = often and 5 = frequently.

#### 2.2.3. Mental Health Outcomes

Depression: Participants filled out the Patient Health Questionnaire (PHQ-9) [29], a common self-reported measure used to screen depressive symptoms. The scale includes nine questions with overall scores ranging from 0 to 27, with scores of 0–4 indicating minimal depression, 5–9 = mild depression, 10–14 = moderate depression, 15–19 = moderately severe depression and ≥20 = severe depression.

Anxiety: anxiety symptoms were measured with the Generalized Anxiety Disorder scale (GAD-7) validated in French [30,31], a reliable and valid seven-questions scale. Each item is scored on a 0–3 scale with overall scores ranging from 0–21. Scores of 0–4 indicate minimal anxiety, 5–9 = mild anxiety, 10–14 = moderate anxiety and 15–21 = severe anxiety.

#### 2.2.4. Covariates

Covariates included in the multivariate analysis are socio-demographic, health-related and COVID-19 related characteristics associated (*p* < 0.20) with social support and loneliness trajectories and mental health outcomes. When potential covariates showed a significant difference between the four data collection waves, an average score was used.

Socio-demographic variables: age (in years), gender (male; female; other), marital status (married/domestic relationship or civil union; in a steady relationship whether cohabitating or not; single; divorced/separated/widowed), number of persons living in the household, area of residence (urban; suburban; rural), years of education, occupation (employed; student; unemployed; retired), change in work frequency due to COVID-19 (no change; change to more/fewer hours; job stopped/lost job; does not apply), income reduction (no reduction; reduction with governmental support; reduction without governmental support), financial worries in the last four weeks (yes vs. no).

Health-related variables: pre-existing mental illnesses (yes vs. no), past-year unhealthy use of tobacco, alcohol and drugs (including illicit drugs and unhealthy use of prescription drug) assessed with the Substance Use Brief Screen (yes vs. no) [32]

COVID-19 related variables: Number of COVID-19 regulations imposed by authorities in the week previous to answering the questionnaire; appropriateness of imposed COVID-19 regulations (disagree; neutral; agree), frequency of going outdoors in the past two weeks (never/rarely; >3 times a week), being quarantined for suspected COVID-19 infection (yes vs. no), knowing someone who has been infected with COVID-19 (yes vs. no), experiencing distress over coronavirus (very little; some; a lot). Individual variables were used for description of the cohort.

### 2.3. Statistical Analyses

First, the study population was described using means and standard deviations for continuous variables and using frequencies and percentages for categorical variables. Differences between respondents and drop-outs on variables of interest between the first and the last study wave were tested using logistic regression. French participants who discontinued participation did not differ significantly from those replying to the different follow-up waves of the COMET study.

Second, trajectories of social support and loneliness were determined using Group-based trajectory modeling (GBTM (PROC TRAJ in SAS 9.4)) [33]. GBTM is a person-centered, semiparametric technique for modeling heterogenous change in longitudinal studies, allowing the identification of different subgroups of individuals sharing similar patterns across time [33]. Missing data are handled under the missing-at-random assumption, where individuals with missing data are assigned to their most likely group. Models were estimated using a censored normal distribution, with the Bayesian information criteria (BIC), entropy and average posterior probabilities (APP ≥ 0.7) of trajectory membership to identify the best-fitting model with the least number of trajectories [34]. For both social support and loneliness, a four-group solution was retained reflecting the most optimal BIC scores and APP (social support BIC −3870.47, APP 0.90 (range 0.86–0.94); loneliness BIC −2913.09, APP 0.82 (range 0.79–0.86)) (see Supplementary Tables S1 and S2 for additional model information). Third, the identified trajectories of social support and loneliness were tested for their longitudinal association with depression and anxiety at wave 4 by means of linear regression analyses with the best functioning trajectories (high social support or low loneliness) serving as reference group. Subsequently, we used multivariable linear regression models to assess associations between trajectories of social support and loneliness and the presence of depression and anxiety symptoms while controlling for previously listed covariates. Rates of missing data on covariates ranged from 0.15% (for distress) to 39.4% (for number of people in household) and were imputed using “Mice” package in R [35]. Excluding individuals with missing data from our analyses did not alter the significance or direction of our results. Finally, to test the robustness of our outcomes, we conducted sensitivity analyses using logistic regression models based on dichotomous cut-offs of mental health outcomes (PHQ-9 > 9, GAD > 9), indicating high levels of depression and anxiety (see Supplementary Table S3). All analyses were conducted using RStudio [36].

## 3. Results

Table 1 shows the characteristics of French participants of the COMET study. Participants were predominantly female (78.6%), with a mean age of 47.5 (± 14.9) years. Most of the sample was married (53.4%) or single (20.2%). On average, participants had 14.5 (± 3.3) years of education and most of the sample was employed at the start of the data collection (73.6%). Financial worries decreased across the pandemic from 24.0% in wave 1 to 17.0% in wave 4, while income stability increased from 75.4% in wave 1 to 89.9% in wave 4. Less than 10% of the participants had been diagnosed with a mental illness throughout the life course. Substance use during the pandemic was highest in wave 1 (i.e., 51.3% for alcohol use) and decreased by wave 4 (i.e., 42.6% for alcohol use). The percentage of individuals considering that COVID-19 related regulations were appropriate decreased across the pandemic from 66% in wave 1 compared to 49% in wave 4.

Frequency of going out more than three days/week was the highest during wave 2 where the sanitary measures were eased up (89.6%), whereas for waves 1, 3 and 4 it was 59.3%, 75.9% and 83.7%, respectively. Most of the study population felt little or some distress related to COVID-19 and less than 8% reported feeling a lot of distress across the four study waves.

### 3.1. Social Support and Loneliness Trajectories

**Figure 1:**
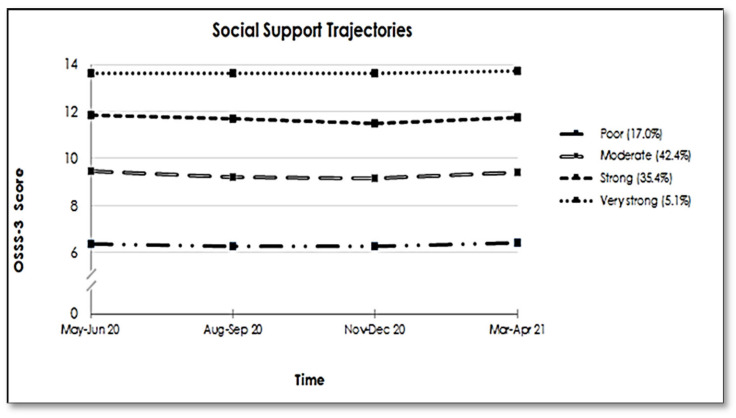
Trajectories of Social Support among the French participants of the COMET study (N = 679).

The social support trajectories (Figure 1) represent perceived social support across the pandemic and reveal four distinct groups in our study population. The two largest groups were those with ‘moderate’ social support (42.4%) (average OSSS-3 score 9.34 ± 0.73) and ‘strong’ support (35.4%) (average OSSS-3 score of 11.74 ± 0.67). In addition, one smaller group (5.1%) showed consistently ‘very strong’ support (average OSSS-3 score 13.66 ± 0.29), while 17.0% belonged to the ‘poor support’ group (average OSSS-3 score 6.28 ± 1.00) over time.

**Figure 2:**
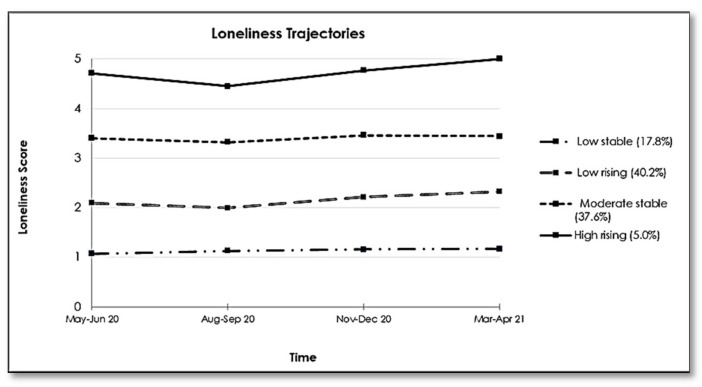
Trajectories of Loneliness among French participants of the COMET study (N = 680).

Regarding the trajectories of loneliness, a four-group model appeared most appropriate (Figure 2). Both a ‘low stable’ group and a ‘moderate stable’ group (comprising 17.8% and 37.6% of the study population, respectively) showed little variation in their average loneliness scores across the pandemic (1.09 ± 0.15 and 3.38 ± 0.44, respectively). The ‘low rising’ group (40.2%; average loneliness score = 2.13 ± 0.35) felt rarely lonely at the start of the study but their feelings of loneliness slowly increased as the pandemic continued. The ‘high rising’ group (5.0%; average loneliness score = 4.78 ± 0.25) reported feeling often/frequently lonely during the first wave, which feelings gradually increased over time, reaching a maximum score of 5 at wave 4, indicating feeling frequently lonely.

### 3.2. Social Support and Loneliness Trajectories and Mental Health Outcomes

#### 3.2.1. Depression

The results of the unadjusted and adjusted linear regression models testing the association between social support and loneliness trajectories and depression are shown in Table 2.

Unadjusted associations between social support trajectories and depression scores (model 1, (adjusted R^2^ = 0.05)) showed that having poor social support was associated with more depressive symptoms (β = 4.12, 95% CI [2.03, 6.20]). Also, in unadjusted model 2 (adjusted R^2^ = 0.30) compared to the low stable loneliness group, participants in all other loneliness trajectories were significantly more likely to have depressive symptoms (low rising β = 2.80, 95% CI [1.54, 4.06]; moderate stable β = 6.00, 95% CI [4.69, 7.31]; high rising β = 12.94, 95% CI [10.72, 15.15]).

When studying both types of trajectories in the same model 3 (adjusted R^2^ = 0.31), associations for social support trajectories were no longer significant; while those for loneliness remained in the same direction as reported above. Finally, when taking additional covariates into account in model 4 (adjusted R^2^ = 0.38), results were slightly attenuated but in the same direction with poor social support (β = 2.04, 95% CI [0.17, 3.91]) and the trajectories of loneliness trajectories being associated with higher levels of depression (low rising β = 1.65, 95% CI [0.36, 2.93]; moderate stable β = 4.51, 95% CI [3.10, 5.92]; high rising β = 10.90, 95% CI [8.61, 13.19]). The results of sensitivity analyses test using the dichotomous PHQ-9 variable as an outcome portrayed the same direction of results (Supplementary Table S3).

#### 3.2.2. Anxiety

Table 2 shows the results of the associations between trajectories of social support, trajectories of loneliness and anxiety. In the first unadjusted regression model (adjusted R^2^ = 0.03), belonging to the poor and moderate social support trajectory group was associated with higher levels of anxiety compared to participants with very strong social support (β = 3.66, 95% CI [1.70, 5.62]; β = 2.26, 95% CI [0.47, 4.05]). Unadjusted analyses in model 2 (adjusted R^2^ = 0.23) regarding loneliness showed all trajectories to be associated with higher levels of symptoms of anxiety (low rising β = 2.05, 95% CI [0.86, 3.25]; moderate stable β = 4.53, 95% CI [3.29, 5.77]; high rising β = 10.10, 95% CI [8.04, 12.15]). Studying both social support and loneliness trajectories at once (model 3, adjusted R^2^ = 0.23), associations between social support trajectories and mental health were no longer significant, while those for loneliness remained in the same direction as reported above. Finally, when adding covariates in model 4 (adjusted R^2^ = 0.36), higher anxiety scores were significantly associated with belonging to either the ‘moderate stable’ loneliness group (β = 2.84, 95% CI [1.59, 4.10]) or ‘high rising’ loneliness group (β = 7.96, 95% CI 5.96, 9.97]). Results of sensitivity analyses using the dichotomous GAD-7 score as an outcome showed results in the same direction as the main analyses (Supplementary Table S3).

## 4. Discussion

To our knowledge, this is the first longitudinal study concomitantly examining trajectories of social support and loneliness across the first year of the COVID-19 pandemic and their associations with mental health outcomes, in a sample of participants from the French general population. We identified four distinct trajectory groups for social support (poor (17.0%), moderate (42.4%), strong (35.4%) and very strong (5.1%)) and loneliness (low stable (17.8%), low rising (40.2%), moderate stable (37.6%) and high rising (5.0%)) between May 2020 and April 2021. Higher levels of depression and anxiety measured after a year were associated with an increasing trajectory of loneliness in particular, while a poor social support trajectory over the pandemic was only associated with depressive symptoms.

### 4.1. Social Support and Loneliness Trajectories

Our analysis of social support trajectories revealed four distinct groups. The majority of our study sample consistently reported having moderate or strong social support that did not seem to be impacted by the pandemic and its associated protective measures such as confinement and social distancing. We also identified a small group of participants (5.0%) who reported very strong social support, and conversely, a proportion of our study population (17.0%) that felt they had poor social support. Both of these groups also remained stable throughout the study period.

Pre-pandemic cross-sectional data from 2014 on perceived social support reported somewhat higher estimates of poor and moderate social support and lower estimates of strong social support in the French population relative to what we reported in our current study [37]. As the prevalence of social support as a whole might have evolved since 2014 it is possible that these numbers do not give an accurate reflection of contemporary levels of perceived social support in the French population. However, the fact that they remained stable might be due to the fact that the population was not impacted by the pandemic. This appears to be supported by another longitudinal study where 260 Chinese participants were followed from pre-COVID, through the peak of COVID-19 and the subsequent decline in cases. While they reported an increase in perceived social support between pre-COVID and the peak of the pandemic, social support remained stable between the second and third waves during the pandemic, which is similar to our results [38].

We also identified four loneliness trajectories, two stable and two rising over time. In France, a longitudinal study reported that around 12% of the population were feeling lonely during the first three months of the pandemic [26]. Another study conducted among 38,200 individuals from the general population in the UK during the first seven weeks of confinement also reported four loneliness trajectories [39] that were more or less similar to the trajectories we identified. While the variation in tools used to assess loneliness has to be taken into consideration, these differences might be due to the difference in the study population with the COMET sample having high percentage of females and educated participants. Most previous studies focused on the early weeks of the pandemic, while our data collection started when some of the imposed restrictive measures in France were eased. In addition, we were able to follow our sample over a longer period of time, including several subsequent lockdown periods, which enabled us to show that loneliness evolves as the pandemic continues over a protracted period of time. While both the ‘low rising’ and ‘high rising’ groups had a small decrease in the reported level of loneliness during the summer period when the sanitary measures were eased up, their average scores subsequently increased in parallel with the second and third confinements. Thus, for some groups in the general population, subsequent confinements might have a cumulative and deleterious impact on their perceived level of loneliness. This is further supported by the observation that we did not identify any decreasing trajectories.

### 4.2. Association between Social Support, Loneliness and Depression and Anxiety

The COVID-19 pandemic and its subsequent sanitary measures (confinements, remote working and social distancing) have been reported in the literature to have an impact on the mental health of many persons. In our study population the prevalence of symptoms of depression ranged between 13.7–24.3% which is closely similar to the repeated cross-sectional data collected in France that reported a peak prevalence of 22.7% during the pandemic [14]. These figures are almost 2.5 times higher than the estimated pre-pandemic prevalence of depressive symptoms (9.8%) [14]. Regarding anxiety, on average 14.6% of our population reported anxiety symptoms, which was slightly higher compared to population data than in the pre-pandemic period (13.5% in 2017) [14].

Results from our adjusted models show that only poor social support was associated with depression symptoms, whereas none of the social support trajectories was significantly associated with anxiety symptoms. While the protective role of perceived social support against anxiety and depressive symptoms has previously been reported [16], the pandemic is a unique period during which in-person social gatherings and interactions have been interrupted except for online social interactions that were maintained. It is possible that study participants preferred not asking for support from friends and relatives over putting them at risk of infection. Likewise, some studies indicate that the protective role of social support is most noticeable when the social support measure is matched with the specific stressor [17], which was not the case in our study. Thus, it is possible that more specific questions on received social support in the context of COVID-19 might have shown a different association with depression or anxiety.

All loneliness trajectories remained associated with depressive and anxiety symptoms in the final adjusted models. The fact that loneliness is associated with depression and anxiety has been reported widely in the literature [25,40] and our outcomes show that this is also the case during the pandemic similar to other studies in the times of COVID-19 [27,41,42]. However, our study goes beyond previous reported results by showing that initial levels of loneliness either remain stable or increase over time during the pandemic and are associated with symptoms of depression and anxiety. In particular, individuals who have high levels of loneliness that increase over time reported the highest levels of anxiety and depression symptoms. It is surprising that social support showed no impact on decreasing the negative impact of the pandemic on feelings of loneliness and its subsequent mental health outcomes, as this has been suggested by previous work reporting on the potential buffering effect of social support [38]. Replicating similar interaction analyses to investigate whether any specific element of social support might have a similar buffering effect was not feasible in our study due to insufficient power. Therefore, whether social support in health emergencies can or cannot counter the effects of loneliness on the mental health remains an area to be further explored in the context of the COVID-19 pandemic.

### 4.3. Strengths and Limitations

Our study has several strengths, among which its longitudinal design. We collected data at four different and equally distant time points (3 during confinement and 1 out of confinement) which provides a good opportunity to understand changes in social support and loneliness throughout the different stages of the pandemic. Furthermore, data were collected in real-time therefore minimizing recall bias. Additionally, we used GBTM to analyze the trajectories of social support and loneliness allowing the identification of different homogeneous subgroups of individuals and observe their variation across the pandemic.

Our study has some limitations as well. First, the COMET study was developed as a response to the emergence of the COVID-19 pandemic and hence, we did not have data collected pre-pandemic to allow us to compare our variables of interest. Therefore, we cannot be sure whether trajectories of social support and loneliness are in reaction to the pandemic, or that they are a continuation of already present subgroups in the population concerning these factors.

Second, our sample was recruited mainly through a snowball sampling method by using social media platforms and universities mailing lists. This method is inexpensive and easy to conduct. However, this might have resulted in selection bias and therefore might hinder the generalizability in terms of prevalence rates. Our sample had an over-representation of females, middle-aged individuals, highly educated and employees whose income was not affected by the crisis. This precludes any inferences regarding the size of the trajectories we found in relation to the general French population, and might have led to an underestimation in our findings regarding the impact on under-represented groups (e.g., people living under precarious circumstances). Likewise, the web-based setting of the study led to non-response on certain items and loss of follow-up mainly between the first and second data wave. It is possible that non-responders to the study or specific items on the questionnaire might have worse mental health outcomes and therefore our findings might be an underestimation. Yet, participants lost to follow-up were not significantly different from those who remained in our study. In addition, our use of a group-based modelling strategy for the estimation of our social support and loneliness trajectories allowed us to include subjects with partial data, thus minimizing the impact of loss-to-follow-up for our study exposure.

Third, although we used validated tools to measure our outcomes, answers were self-reported which might generate information bias. However, it is difficult to estimate to what extent this affected our results given our study was largely focused on mental health in COVID-19, a situation of which epidemiological information is still inconclusive. In addition, the loneliness questionnaire was composed of only one question. While this type of question has been used in other studies, it assumes a common understanding of “loneliness” among the participants specially that we are measuring the perceived loneliness and not the actual social isolation [42]. Nevertheless, using a single item to measure loneliness is an accepted practice in large scale surveys and this approach has been shown to be highly correlated with multidimensional scales like the University of California Los Angeles (UCLA) loneliness scale [43] and the De Jong Gierveld Loneliness scale [44], which suggests that the single-item approach does indeed capture some aspects of loneliness [45].

Finally, the study sample size does not allow us to conduct further stratified analysis to observe, for example, the differences in outcomes according to socio-demographic indicators. However, it is possible to see whether our outcomes can be replicated for the whole COMET cohort, which includes 8084 persons and gives more statistical power to detect small differences

### 4.4. Future Implications

Our findings revealed an association between poor social support, as well as high and increasing levels of loneliness and symptoms of depression and anxiety during the first year of the COVID-19 pandemic in France. These outcomes strongly suggest the necessity for strategizing and implementing interventions designed to combat loneliness. Interventions can be integrated at the societal and individual levels: First, at the societal level, raising awareness on the risks of loneliness and the importance of social relationships and putting in place policies including everyone at risk, for example, ensuring the continuous screening and early detection of loneliness and mental health symptoms [46]. Furthermore, it is utterly important to balance the necessity of lockdowns and therefore decreasing the propagation of the virus, with the negative effects these lockdowns carry on an individual’s feeling of loneliness and subsequent mental health outcomes. Increasing vaccination coverage in the population might be the tool to restrict the necessity of lockdowns similar to ones experienced in the past year. Second at the individual level, several low-cost, COVID-19 friendly interventions have been reported in the literature as effective in decreasing the feelings of loneliness like online Cognitive Behavioral Therapy (CBT) and online mindfulness exercises. Mindfulness exercises, which are meditation-based exercises, have also been shown to decrease levels of loneliness in different age groups [47] and in enhancing relationships [48]. A randomized control trial testing online intervention for loneliness in older persons has proven to be effective in times of COVID-19 [49]. Finally, given the underrepresentation of certain groups in our study population (e.g., those living in precarious conditions), it important to replicate the study in these groups, to identify the specific supporting factors that are relevant for them, as well as the impact of these factors on their mental health outcomes.

## 5. Conclusions

The observed outcomes in our study showed that in particular persons with high and increasing levels of loneliness during the COVID-19 pandemic in France have elevated symptoms of anxiety and depression. The pandemic has not ended yet, and sanitary measures are still being adapted according to the situation; therefore, it remains essential to monitor potentially vulnerable populations and combat loneliness in order to mitigate negative consequences on mental health.

## Figures and Tables

**Table 1:** Descriptive Statistics for French participants in the COMET Cohort.

	Wave 1	Wave 2	Wave 3	Wave 4	p *
	N = 681	N = 442	N = 441	N = 424	
***Socio-demographic***					
Age (in years)	46.49 (14.92)	---	---	---	
Gender					0.788
Female	531 (78.6)	354 (80.9)	346 (79.0)	337 (80.2)	
Male	145 (21.4)	84 (19.1)	92 (21.0)	83 (19.8)	
Residential Area					<0.001
Urban	346 (52.0)	---	---	223 (53.6)	
Suburban	137 (20.6)	---	---	90 (21.6)	
Rural	182 (27.4)	---	---	103 (24.8)	
Relationship Status					
Single	137 (20.2)	90 (20.6)	---	75 (17.9)	0.884
Married, domestic relationship or civil union	363 (53.4)	238 (54.3)	---	230 (55.0)	
In a steady relationship (cohabitating or not)	99 (14.6)	54 (12.3)	---	58 (13.9)	
Divorced, separated, widowed	81 (11.9)	56 (12.9)	---	55 (13.2)	
Number of people in household	2.52 (1.25)	2.14 (0.88)	2.47 (1.22)	2.45 (1.22)	0.593
Education (in years)	14.52 (3.25)	---	---	---	
Occupation					<0.001
Student	39 (5.8)	---	---	15 (3.6)	
Worker	499 (73.6)	---	---	299 (71.7)	
Unemployed	77 (11.4)	---	---	49 (11.8)	
Retired	63 (9.3)	---	---	54 (13.0)	
Change in work frequency					<0.001
No change	218 (32.2)	117 (26.7)	160 (37.2)	140 (33.6)	
Change (more/less)	280 (41.3)	206 (47.0)	162 (37.67)	152 (36.4)	
Job stopped/lost job	41 (6.1)	22 (5.0)	17 (4.0)	17 (4.1)	
Does not apply	139 (21.0)	93 (21.2)	91 (21.2)	108 (25.9)	
Income reduction/government support					<0.001
Neither	506 (75.4)	377 (85.7)	378 (87.7)	373 (89.9)	
Income reduced/support	44 (6.6)	12 (2.7)	12 (2.8)	3 (0.7)	
Income reduced/no support	121 (18.0)	51 (11.6)	41 (9.5)	39 (9.4)	
Financial worries (yes)	162 (24.0)	87 (19.8)	71 (16.8)	70 (17.0)	0.008
***Health***					
Mental Illness (yes)	67 (9.9)	---	---	36 (8.6)	<0.001
Substance use (yes)					
Tobacco	186 (27.6)	---	---	103 (24.7)	<0.001
Alcohol	346 (51.3)	---	---	177 (42.6)	<0.001
Medications recreationally/illegal drug	77 (11.4)	---	---	33 (7.9)	<0.001
***COVID-19 related factors***					
Number of COVID-19 regulations imposed	7.55 (3.14)	5.74 (2.66)	6.97 (2.89)	6.58 (2.23)	<0.001
Considers regulations appropriate					<0.001
Disagree	137 (20.3)	---	---	146 (35.0)	
Neutral	90 (13.3)	---	---	68 (16.3)	
Agree	449 (66.4)	---	---	203 (48.7)	
Frequency of going outdoors					<0.001
Never/rarely	275 (40.7)	46 (10.5)	104 (24.1)	68 (16.3)	
3 days/week	400 (59.3)	394 (89.6)	327 (75.9)	349 (83.7)	
Quarantine for (suspected) COVID-19 (yes)	47 (7.0)	42 (9.7)	---	---	<0.001
Know someone who’s been infected by COVID-19 (yes)	486 (71.8)	312 (70.8)	355 (82.4)	365 (87.5)	<0.001
Experienced distress related to the coronavirus					0.006
Very little	346 (50.9)	265 (60.5)	239 (55.5)	198 (47.6)	
Some	286 (42.1)	150 (34.3)	159 (36.9)	185 (44.5)	
A lot	48 (7.1)	23 (5.3)	33 (7.7)	33 (7.9)	
Social support score	9.97 (2.36)	9.80 (2.48)	9.80 (2.46)	10.00 (2.47)	0.395
Loneliness score	2.54 (1.18)	2.46 (1.15)	2.58 (1.17)	2.66 (1.19)	0.109
Patient Health Questionnaire (PHQ-9) score	6.38 (5.63)	4.78 (4.87)	6.45 (5.48)	6.07 (5.25)	<0.001
Depression score >9	158 (24.0)	58 (13.7)	102 (24.3)	87 (21.5)	<0.001
Generalized Anxiety Disorder scale (GAD-7) score	4.81 (4.93)	4.06 (4.55)	4.97 (4.67)	4.76 (4.73)	0.022
Anxiety score >9	104 (15.6)	48 (11.1)	70 (16.5)	63 (15.4)	0.108

Numbers are represented as n (%) for categorical variables or mean (S.D.) for continuous variables. (---) data not collected during the wave; *p* *: *p*-value indicating the response differences between each data wave; Wave 1: 4 May–7 July 2020; wave 2: 4 September–5 October 2020; wave 3: 7 December 2020–10 January 2021; wave 4: 19 March–23 April 2021.

**Table 2:** The association of social support and loneliness trajectories with symptoms of depression (n = 364) and anxiety (n = 369) in French participants of the COMET cohort.

Depressive Symptoms					
		Model 1	Model 2	Model 3	Model 4 ^§^
		β [95% CI]	β [95% CI]	β [95% CI]	β [95% CI]
Social	Poor	4.12 [2.03, 6.20] ***		1.86 [−0.06, 3.78]	2.04 [0.17, 3.91] *
Support	Moderate	1.55 [−0.38, 3.48]		0.28 [−1.47, 2.02]	0.30 [−1.37, 1.96]
Trajectories	Strong	1.05 [−0.85, 2.95]		0.89 [−0.86, 2.65]	0.97 [−0.70, 2.64]
	Very strong	-Ref-		-Ref-	-Ref-
Loneliness Trajectories	Low stable		-Ref-	-Ref-	-Ref-
	Low rising		2.80 [1.54, 4.06] ***	2.79 [1.52, 4.06] ***	1.65 [0.36, 2.93] *
	Moderate stable		6.00 [4.69, 7.31] ***	5.84 [4.50, 7.18] ***	4.51 [3.10, 5.92] ***
	High rising		12.94 [10.72, 15.15] ***	12.50 [10.25, 14.75] ***	10.90 [8.61, 13.19] ***
Model Adjusted R^2^		0.05	0.3	0.31	0.38
**Symptoms of Anxiety**					
		**Model 1**	**Model 2**	**Model 3**	**Model 4 ^&^**
		**β [95% CI]**	**β [95% CI]**	**β [95% CI]**	**β [95% CI]**
Social	Poor	3.66 [1.70, 5.62] ***		1.37 [−0.44, 3.18]	1.06 [−0.63, 2.76]
Support	Moderate	2.26 [0.47, 4.05] *		0.86 [−0.79, 2.50]	0.62 [−0.89, 2.13]
Trajectories	Strong	1.79 [−0.03, 3.61]		1.19 [−0.46, 2.84]	1.04 [−0.47, 2.55]
	Very strong	-Ref-		-Ref-	-Ref-
Loneliness Trajectories	Low stable		-Ref-	-Ref-	-Ref-
	Low rising		2.05 [0.86, 3.25] **	1.93 [0.71, 3.14] **	0.80 [−0.37, 1.96]
	Moderate stable		4.53 [3.29, 5.77] ***	4.35 [3.07, 5.64] ***	2.84 [1.59, 4.10] ***
	High rising		10.10 [8.04, 12.15] ***	9.87 [7.76, 11.98] ***	7.96 [5.96, 9.97] ***
Model Adjusted R^2^		0.03	0.23	0.23	0.36

Models 1 and 2: unadjusted linear regression between social support (1), loneliness trajectories (2) and depression or anxiety symptoms; Model 3: Unadjusted linear regression between social support and loneliness trajectories and depression or anxiety symptoms; ^§^ model adjusted for: Age, gender, education, relationship status, knowing someone with COVID-19, diagnosed mental illness, distress related to COVID-19 pandemic; ^&^ model adjusted for: Age, gender, number of people in the household, consider the COVID-19 regulation appropriate, mental illness, financial worries distress related to COVID-19 pandemic; (***) *p*-value < 0.001; (**) *p*-value < 0.01; (*) *p*-value < 0.05; β = linear regression coefficient; CI = confidence interval.

## Data Availability

The data underlying the findings cannot be made freely available because of ethical and legal restrictions. This is because the present study includes an important number of variables that, together, could be used to re-identify the participants based on a few key characteristics and then be used to have access to other personal data. Therefore, the French ethical authority strictly forbids making such data freely available. However, they can be obtained upon request from the COMET consortium to request access.

## Supplementary Materials

The following are available online at https://www.mdpi.com/article/10.3390/ijerph182312677/s1, Table S1: Identification of trajectory models of social support trajectories, Table S2: Identification of trajectory models of loneliness trajectories, Table S3: The association of social support and loneliness trajectories with symptoms of depression and anxiety in French participants of the COMET cohort- logistic regression analysis.
