# Supplementary material for: Impact of Longitudinal Social Support and Loneliness Trajectories on Mental Health during the COVID-19 Pandemic in France
Source: Int J Environ Res Public Health. 2021 Dec 1;18(23):12677. doi: 10.3390/ijerph182312677 (PMC8656819; doi:10.3390/ijerph182312677)
Supplement: Supplementary file 1 [file ijerph-18-12677-s001.zip › ijerph-1473634-supplementary.pdf]

Supplementary Materials

**Table S1.** Identification of trajectory models of social support trajectories				
	BIC	2ΔBIC	Entropy	Sample percentage per class membership
1st	-4491.16	-Ref-	1.00	100.0
2nd	-4127.84	726.64	0.90	40.0/60.0
3rd	-3939.9	1102.52	0.91	18.3/48.7/33.0
4th	**-3870.47**	**1241.38**	0.90	17.4/41.0/35.9/5.7
5th	-3863.16	1256	0.86	6.9/13.8/39.4/34.3/5.7
6th	-3867.5	1247.32	0.79	6.0/12.4/16.6/27.9/31.7/5.4
BIC = Bayesian information Criterion				

**Table S2.** Identification of trajectory models of loneliness trajectories				
	BIC	2ΔBIC	Entropy	Percentage sample Size per class membership
1st	-3268.92	-Ref-	1.00	100.0
2nd	-3036.48	464.88	0.92	30.7/69.3
3rd	-2934.54	668.76	0.90	22.5/64.5/13.0
4th	**-2913.09**	**711.66**	0.82	17.0/42.5/35.5/5.0
5th	-2918.9	<u>700.04</u>	0.82	16.8/39.7/36.4/3.5/3.6
6th	-2914.86	708.12	0.80	12.6/6.5/37.7/35.1/3.6/4.5

Table S3. The association of social support and loneliness trajectories with clinical levels of depression (n=364) and anxiety (n=369) in French participants of the COMET cohort- logistic regression analysis

Depressive symptoms (PHQ-9 >9)					
		**Model 1**	**Model 2**	**Model 3**	**Model 4§**
		OR [95% CI]	OR [95% CI]	OR [95% CI]	OR [95% CI]
**Social Support Trajectories**	Poor	**5.67 [1.57, 20.49]****		2.69 [0.66, 10.90]	3.28 [0.70, 15.40]
	Moderate	1.96 [0.56, 6.93]		1.14 [0.29, 4.49]	1.20 [0.27, 5.32]
	Strong	2.09 [0.59, 7.44]		1.74 [0.44, 6.84]	1.94 [0.44, 8.55]
	Very strong	-Ref-		-Ref-	-Ref-
**Loneliness Trajectories**	Low stable		-Ref-	**-Ref-**	**-Ref-**
	Low rising		**5.05 [1.16, 22.03]*****	**5.12 [1.16, 22.56]***	2.73 [0.59, 12.61]
	Moderate stable		**14.60 [3.40, 62.68]*****	**14.03 [3.22, 61.11]*****	**8.09 [1.75, 37.42] ****
	High rising		**71.17 [12.90, 392.55]*****	**62.96 [11.15, 355.40} *****	**36.63 [5.70, 235.27] *****
Symptoms of Anxiety (GAD>9)					
		**Model 1**	**Model 2**	**Model 3**	**Model 4&**
		OR [95% CI]	OR [95% CI]	OR [95% CI]	OR [95% CI]
**Social Support Trajectories**	Poor	**9.64 [1.22, 76.27]***		4.72 [0.52, 43.17]	8.01 [0.54, 118.30]
	Moderate	5.36 [0.69, 41.34]		3.64 [0.41, 32.15]	6.64 [0.47, 93.61]
	Strong	4.71 [0.60, 36.83]		4.62 [0.52, 41.32]	8.04 [0.56, 115.07]
	Very strong	-Ref-		-Ref-	-Ref-
**Loneliness Trajectories**	Low stable		-Ref-	-Ref-	-Ref-
	Low rising		7.10 [0.93, 54.44]	6.39 [0.83, 49.27]	2.57 [0.31, 21.21]
	Moderate stable		**16.05 [2.12, 121.38] ****	**14.23 [1.86, 108.69] ***	5.54 [0.68, 45.36]
	High rising		**124 [14.02, 1097.06]*****	**113.08 [12.49, 1023.75]*****	**60.38, [6.04, 603.17]*****

Model 1&2: unadjusted logistic regression between social support (1), loneliness trajectories (2) and clinical levels of depression or anxiety
Model 3: unadjusted logistic regression between social support and loneliness trajectories and clinical levels of depression or anxiety
§Model adjusted for: age, gender, education, relationship status, knowing someone with COVID-19, diagnosed mental illness, distress related to COVID-19 pandemic;
& Model adjusted age, gender, number of people in the household, consider the COVID-19 regulation appropriate, mental illness, financial worries distress related to COVID-19 pandemic
(***) p-value<0.001; (**) p-value<0.01; (*) p-value<0.05; OR = Odds ratio;
CI = confidence interval.
